# Biomarkers for ragwort poisoning in horses: identification of protein targets

**DOI:** 10.1186/1746-6148-4-30

**Published:** 2008-08-08

**Authors:** Rowan E Moore, Derek Knottenbelt, Jacqueline B Matthews, Robert J Beynon, Phillip D Whitfield

**Affiliations:** 1 Proteomics and Functional Genomics Research Group, Faculty of Veterinary Science, University of Liverpool, Crown Street, Liverpool, L69 7ZJ, UK; 2 Equine Division, Faculty of Veterinary Science, University of Liverpool Veterinary Teaching Hospital, Leahurst, Neston, CH64 7TE, UK; 3 Division of Parasitology, Moredun Research Institute, Midlothian, EH26 OPZ, UK

## Abstract

**Background:**

Ingestion of the poisonous weed ragwort (*Senecio jacobea*) by horses leads to irreversible liver damage. The principal toxins of ragwort are the pyrrolizidine alkaloids that are rapidly metabolised to highly reactive and cytotoxic pyrroles, which can escape into the circulation and bind to proteins. In this study a non-invasive *in vitro *model system has been developed to investigate whether pyrrole toxins induce specific modifications of equine blood proteins that are detectable by proteomic methods.

**Results:**

One dimensional gel electrophoresis revealed a significant alteration in the equine plasma protein profile following pyrrole exposure and the formation of a high molecular weight protein aggregate. Using mass spectrometry and confirmation by western blotting the major components of this aggregate were identified as fibrinogen, serum albumin and transferrin.

**Conclusion:**

These findings demonstrate that pyrrolic metabolites can modify equine plasma proteins. The high molecular weight aggregate may result from extensive inter- and intra-molecular cross-linking of fibrinogen with the pyrrole. This model has the potential to form the basis of a novel proteomic strategy aimed at identifying surrogate protein biomarkers of ragwort exposure in horses and other livestock.

## Background

Ragwort (*Senecio jacobea*) is a poisonous weed found growing on riverbanks, roadsides and pasture and is toxic to most grazing livestock. Horses are particularly sensitive to the toxic effects of ragwort and typically avoid the weed, but poisoning can occur when the animals graze on poorly maintained pastures or when feedstuff such as hay or silage is contaminated. Ragwort poisoning leads to complete liver failure, however the clinical signs can be slow to develop and may only manifest after prolonged dietary exposure. Prognosis is poor once the liver disease is advanced and current treatments are only palliative [1]. The early diagnosis of ragwort poisoning is extremely difficult as many conventional biochemical and histopathological indicators of the disease are non-specific and do not discriminate ragwort poisoning from other immune, infectious or toxic diseases [2].

**Figure 1:**
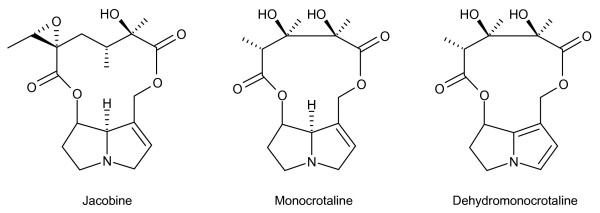
**Chemical structure of pyrrolizidine alkaloids**. Jacobine is one of the major pyrrolizidine alkaloids found in ragwort. The related pyrrolizidine alkaloid, monocrotaline was used in the experimental procedures of this study. Monocrotaline was chemically oxidised to its pyrrolic derivative, dehydromonocrotaline (DHM) and exposed to equine plasma proteins.

The toxic precursors in ragwort are pyrrolizidine alkaloids [3-5]. Amongst the most prominent pyrrolizidine alkaloids found in ragwort are jacobine, erucifoline and senecionine [6,7]. Following ingestion of ragwort the pyrrolizidine alkaloids are absorbed from the gastrointestinal tract and pass to the liver where they can be rapidly metabolised by the cytochrome P450 system to reactive and cytotoxic pyrroles [8,9] (Figure 1). The pyrrolic metabolites bind to liver macromolecules such as DNA, eliciting cell injury [10,11] but they can also escape into the circulation where they react with the thiol groups of cysteine residues of blood proteins [12,13]. The persistence of these adducts formed the basis of one chemical analysis that was developed over a decade ago [14,15]. In this test, haemoglobin thioesters were treated with ethanolic silver nitrate under acidic conditions, releasing a diethoxyether form of the bound pyrrole that can be identified by thin-layer chromatography, gas chromatography or high performance liquid chromatography. This approach has been used to confirm ragwort poisoning in horses [16], cattle [17] and yaks [18] but has not been adopted widely.

An alternative strategy might be to directly analyse pyrrole-protein complexes in blood rather than by the recovery of the xenobiotics. In this study an *in vitro *model system has been developed to investigate whether pyrrole toxins induce specific modifications of equine blood proteins that are detectable by proteomic methods. Such an approach may identify target proteins that could serve as surrogate markers of ragwort exposure. It offers the possibility of a novel analytical strategy to improve the diagnosis of ragwort toxicity in horses and other livestock.

## Results and discussion

Ethical considerations preclude toxicological studies that would require horses to be exposed to ragwort. Therefore, an *in vitro *model system was developed to determine the modifications to horse blood proteins induced by the toxic metabolites of ragwort. Commercial preparations of the major pyrrolizidine alkaloids from ragwort are expensive and therefore a structurally and chemically related pyrrolizidine alkaloid, monocrotaline, was used to optimise methodologies. The model involved the chemical oxidation of monocrotaline to its pyrrolic derivative, dehydromonocrotaline (DHM), followed by modification of horse proteins obtained from surplus clinical blood samples taken for diagnostic purposes. Proteomic methods were then used to detect specific alterations of the equine blood proteins.

### Pyrrole modification of equine haemoglobin

Pyrrole thioesters of haemoglobin are believed to be relatively stable [12-14] and may remain in the bloodstream for the lifespan of the erythrocyte (approximately four months in the horse). The detection of pyrrole-modified haemoglobin molecules could act as an indicator of ragwort exposure over a prolonged period. Equine haemoglobin, prepared from freshly collected blood, was incubated *in vitro *with varying concentrations of synthesised DHM and analysed for evidence of pyrrole modification. 1-D SDS-PAGE showed no changes in the profile of the haemoglobin following pyrrole exposure and simply separated the α- and β-chains. This approach was unlikely to provide sufficient resolution to discriminate between modified and non-modified haemoglobin molecules if a single cysteine residue had been adducted. The haemoglobin molecule was further analysed by intact protein mass spectrometry. Free cysteine and cysteine-containing peptides such as glutathione are readily modified by pyrrolic metabolites [19-21]. The adduction of a pyrrole to a cysteine residue would be expected to increase the molecular weight of each haemoglobin chain by either 117 or 135 Da, depending on whether the pyrrole moiety dehydrates. Intact mass analysis of the haemoglobin using electrospray ionisation-mass spectrometry (ESI-MS) revealed two peaks at 15,114 ± 2 Da and 16,008 +/- 2 Da, which corresponded to the predicted molecular weights of the α- and β-chains respectively. An additional peak at 15,098 +/- 2 Da was due to a Phe_24_→Tyr_24 _variant of the α-chain equine haemoglobin, whilst a peak detected at 16,313 +/- 2 Da was attributed to the glutathionylated form of the β-chain, indicating that the cysteine residue was accessible in a significant proportion of the β-chain. The haemoglobin was also digested with trypsin and the resultant peptides were analysed by matrix assisted laser desorption ionisation-time-of-flight-mass spectrometry (MALDI-ToF-MS). Despite 90% sequence coverage of each of the globin chains, the analysis failed to reveal any pyrrole modification of the cysteine containing peptide.

The inability to detect pyrrole adducts may be due to a combination of factors. The pyrrole modification may be spread over the α- and β-chains of haemoglobin and it is possible that the extent of modification of haemoglobin is too low to be observed in the predominant unmodified haemoglobin pool. It is also possible that glutathione in the erythrocytes may have protected the haemoglobin from pyrrole modification, however glutathione-pyrrole adducts were not detected in blood samples by mass spectrometric analysis. In addition, there may be difficulties in reacting pyrroles with intact equine haemoglobin. The α-chain cysteine residue of horse haemoglobin is situated in a relatively hydrophobic region of the protein, which is likely to render it inaccessible to pyrrole metabolites. However, the β-chain cysteine at position 93 is more externally located and is more susceptible to modification [22,23]. This conjecture was confirmed when equine haemoglobin was modified with the pyrrole under reducing and denaturing conditions and a small amount of β-chain adduction was detected by intact mass analysis using ESI-MS.

### Pyrrole modification of equine plasma proteins

**Figure 2:**
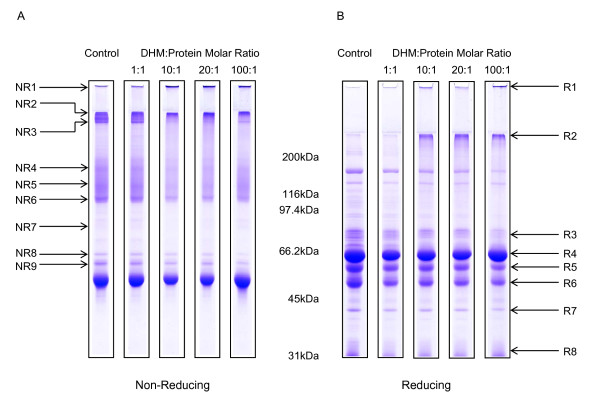
**The effect of pyrrole exposure on equine plasma protein profile**. Equine plasma was reacted with 10–100 molar excess of DHM for 60 min at room temperature. Control horse plasma was treated with a reaction mixture that was complete apart from DHM. Proteins were separated by 1-D SDS-PAGE on 7.5% (w/v) gels under (A) non-reducing and (B) reducing conditions. Gels were stained with Coomassie brilliant blue. High molecular weight aggregates are evident in pyrrole treated lanes from both gels. Proteins identifications are detailed in Tables 1 and 2.

Other potential protein targets of pyrrole modification in horse blood were also investigated. As the first site of exposure to pyrroles would be plasma proteins, equine plasma was incubated with chemically synthesised DHM and the proteins analysed by 1-D SDS-PAGE. Both non-reducing and reducing gels revealed a significant alteration in the equine plasma protein profile following pyrrole exposure (Figure 2). There was a shift in mobility of a number of protein bands and a protein aggregate was detected in the high molecular weight region of the resolving gel, which became more pronounced with increasing concentrations of DHM. The aggregate was not observed in control samples where plasma was treated with a reaction mixture that was complete apart from DHM. Similarly, controls where monocrotaline or the carrier solvent, N,N-dimethylformamide (DMF), was exposed to plasma also failed to produce any changes in the protein profile.

Modification was therefore caused by the presence of pyrrole in the experimental system. DHM is capable of cross-linking proteins [24,25]. The proteins in the high molecular weight aggregate at the top of the gel were identified using liquid chromatography-tandem mass spectrometry (LC-MS/MS). The major components of the aggregate were identified with high confidence (*p *≤ 0.05) as serum albumin, fibrinogen, transferrin and immunoglobulin gamma (IgG) (Tables 1 and 2).

**Table 1:** Identification of proteins from equine plasma in non-reducing 1-D SDS-PAGE. The plasma proteins were identified using LC-MS/MS.

Band No.	Protein Identification	Accession No.	*M*_r _(kDa)	p*I*	MOWSE Score ^a ^(*p *≤ 0.05)	No. of Peptides Matched	% Coverage^b^	Species
NR1	Serum albumin	CAA52194	70.6	5.95	403 (44)	41	34	*Equus caballus*
	Fibrinogen A α chain (fragment)	AAC67561	49.6	6.45	138 (44)	11	7	*Equus caballus*
	Immunoglobulin gamma 4 heavy chain constant region (fragment)	CAC44763	37.0	8.04	147 (44)	15	20	*Equus caballus*
	Immunoglobulin gamma 7 heavy chain	AAS18414	36.3	7.70	109 (44)	13	-	*Equus caballus*
	Immunoglobulin gamma 4 heavy chain	AAS18415	36.2	7.71	106 (44)	15	-	*Equus caballus*
	Transferrin	AAA63684	80.3	6.83	102 (44)	5	6	*Equus caballus*

NR2	Serum albumin	CAA52194	70.6	5.95	477 (45)	34	41	*Equus caballus*
	Immunoglobulin mu	AAU09792	50.1	5.52	372 (45)	24	44	*Equus caballus*
	Lambda immunoglobulin (fragment)	AAA50978	17.7	8.35	165 (45)	9	31	*Equus caballus*
	Fibronectin	AAC48614	58.1	5.91	124 (45)	3	7	*Equus caballus*

NR3	Fibrinogen A α chain (fragment)	AAC67561	49.6	6.45	754 (44)	42	45	*Equus caballus*
	Serum albumin	CAA52194	68.6	5.95	265 (44)	22	24	*Equus caballus*
	Fibrinogen γ B chain	CAA33562	50.8	5.54	121 (44)	6	6	*Bos taurus*

NR4	Lamba Immunoglobulin (fragment)	AAA50978	17.4	8.35	274 (44)	15	32	*Equus caballus*
	Immunoglobulin gamma 5 heavy chain constant region (fragment)	CAC86340	35.9	5.95	272 (44)	20	28	*Equus caballus*
	Immunoglobulin gamma 1 heavy chain constant region (fragment)	CAC44624	37.4	7.68	270 (44)	7	17	*Equus caballus*
	Serum albumin	CAA52194	68.6	5.95	265 (44)	22	24	*Equus caballus*

NR5	Immunoglobulin gamma 1 heavy chain constant region (fragment)	CAC44624	37.4	7.68	554 (44)	26	34	*Equus caballus*
	Immunoglobulin G heavy chain (fragment)	AAG01011	47.6	5.70	411 (44)	30	27	*Equus caballus*

NR6	Serum albumin	CAA52194	70.6	5.95	717 (44)	59	48	*Equus caballus*
	Immunoglobulin gamma 4 heavy chain	AAS18415	36.2	7.71	463 (44)	59	-	*Equus caballus*
	Immunoglobulin gamma 7 heavy chain	AAS18414	36.3	7.70	454 (44)	56	-	*Equus caballus*
	Immunoglobulin gamma 4 heavy chain constant region (fragment)	CAC44763	37.0	8.04	374 (44)	46	37	*Equus caballus*
	Lambda immunoglobulin (fragment)	AAA50978	7.7	8.35	337(44)	52	-	*Equus caballus*

NR7	Gelsolin	Q28372	80.9	5.58	354(44)	15	20	*Equus caballus*

NR8	Transferrin	AAA63684	80.3	6.83	421 (44)	31	27	*Equus caballus*
	Carboxylesterase 1	AAH21150	63.0	5.64	114 (44)	6	6	*Mus musculus*

NR9	Transferrin	AAA63684	80.3	6.83	665 (44)	51	32	*Equus caballus*
	α-1-antitrypsin	AAC83412	47.1	5.23	150 (44)	7	8	*Equus caballus*
	Serum albumin	AAV28861	70.5	5.89	95 (44)	8	12	*Equus asinus*

Proteins were identified on the basis of their MOWSE score using the MSDB database. Each band number refers to proteins identified in Figure 2A. ^a ^= MOWSE baseline significance score in brackets; ^b ^= for those with no percentage coverage value the database did not give this information.

**Table 2:** Identification of proteins from equine plasma in reducing 1-D SDS-PAGE.

Band No.	Protein Identification	Accession No.	*M*_r _(kDa)	p*I*	MOWSE Score^a ^ (*p*≤ 0.05)	No. of Peptides Matched	% Coverage^b^	Species
R1	Serum albumin	CAA52194	70.5	5.95	437 (44)	48	30	*Equus caballus*
	Immunoglobulin gamma 7 heavy chain	AAS18414	36.3	7.70	132 (44)	8	-	*Equus caballus*
	Immunoglobulin gamma 4 heavy chain	AAS18415	36.2	7.71	127 (44)	10	-	*Equus caballus*
	Fibrinogen A α chain (fragment)	AAC67561	49.6	6.45	49 (45)	3	7	*Equus caballus*

R2	Serum albumin	CAA52194	70.6	5.95	639 (44)	39	31	*Equus caballus*
	Fibrinogen A α chain (fragment)	AAC67561	49.6	6.45	230 (44)	14	19	*Equus caballus*
	Immunoglobulin G heavy chain (fragment)	AAG01011	47.6	5.7	141 (44)	8	14	*Equus caballus*
	Immunoglobulin G light chain (fragment)	AAG01010	24.5	6.4	141 (44)	4	9	*Equus caballus*
	Apolipoprotein A-1	P02648	30.2	5.2	94 (44)	6	7	*Canis familiaris*

R3	Transferrin	AAA63684	80.3	6.83	685 (44)	47	32	*Equus caballus*
	Fibrinogen A α chain	AAC67561	49.6	6.45	195 (44)	15	26	*Equus caballus*
	Serum albumin	CAA52194	70.6	5.95	187 (44)	16	14	*Equus caballus*
	Immunoglobulin mu	AAU09792	50.1	5.52	127 (44)	10	16	*Equus caballus*

R4	Serum albumin	AAV28861	70.5	5.89	249 (45)	18	16	*Equus caballus*
	Fibrinogen A α chain	AAC67561	49.6	6.45	50 (45)	3	5	*Equus caballus*

R5	Immunoglobulin gamma 7 heavy chain	AAS18414	36.3	7.7	305 (44)	25		*Equus caballus*
	Immunoglobulin gamma 4 heavy chain constant region (fragment)	CAC44763	37.0	8.04	280 (44)	24	32	*Equus caballus*
	Immunoglobulin gamma 5 heavy chain constant region (fragment)	CAC86340	36.4	5.95	256 (44)	24	33	*Equus caballus*
	Immunoglobulin G heavy chain (fragment)	AAG01011	47.6	5.7	256 (44)	22	21	*Equus caballus*
	Immunoglobulin gamma 4 heavy chain	AAS18415	36.2	7.71	247 (44)	24	-	*Equus caballus*
	Immunoglobulin gamma 1 heavy chain constant region (fragment)	CAC44624	38.0	7.68	224 (44)	11	19	*Equus caballus*
	Immunoglobulin gamma 6 heavy chain constant region (fragment)	CAC86341	36.5	8.03	212 (44)	13	24	*Equus caballus*
	Serum albumin	CAA52194	70.6	5.95	92 (44)	6	4	*Equus caballus*
	Haptoglobin	CAA25267	39.0	6.13	75 (45)	5	-	*Homo sapiens*

R6	Immunoglobulin gamma 7 heavy chain	AAS18414	36.3	7.7	305 (44)	25		*Equus caballus*
	Immunoglobulin gamma 4 heavy chain constant region (fragment)	CAC44763	37.0	7.28	280 (44)	24	32	*Equus caballus*
	Immunoglobulin gamma 4 heavy chain	AAS18415	36.2	7.71	247 (44)	24	-	
	Immunoglobulin gamma 1 heavy chain constant region (fragment)	CAC44624	38.0	7.68	224 (44)	11	19	*Equus caballus*
	Immunoglobulin gamma 6 heavy chain constant region (fragment)	CAC86341	36.5	8.03	212 (44)	13	24	*Equus caballus*

R7	Immunoglobulin gamma 4 heavy chain	AAS18415	36.2	7.71	93 (44)	3	10	*Equus caballus*
	Immunoglobulin gamma 4 heavy chain constant region (fragment)	CAC44763	37.0	7.28	93 (44)	3	10	*Equus caballus*

R8	Apolipoprotein A-1	P02648	30.1	5.2	608 (44)	36	27	*Canis familiaris*
	Lambda immunoglobulin (fragment)	AAA50978	17.6	8.35	295 (44)	17	23	*Equus caballus*
	Lambda immunoglobulin (fragment)	AAA50971	20.7	8.22	192 (44)	7	24	*Equus caballus*

The plasma proteins were identified using LC-MS/MS. Proteins were identified on the basis of their MOWSE score using the MSDB database. Each band number refers to proteins identified in Figure 2B. ^a ^= MOWSE baseline significance score in brackets; ^b ^= for those with no percentage coverage value the database did not give this information.

Due to the evidence of cross-linking of the major plasma proteins authentic standards of serum albumin, fibrinogen and transferrin were reacted with DHM. As equine protein standards were not available from commercial sources bovine standards were used in their place. Fibrinogen appeared to be particularly susceptible to modification by the pyrrole, being substantially converted to a high molecular weight aggregate. In contrast, the bovine serum albumin and transferrin standards displayed only limited reactivity with DHM and did not show the same shift in mobility corresponding to the high molecular weight aggregate. It was possible that the presence of IgG, albumin and transferrin in whole plasma was due to co-polymerisation and aggregation with the fibrinogen molecule.

**Figure 3:**
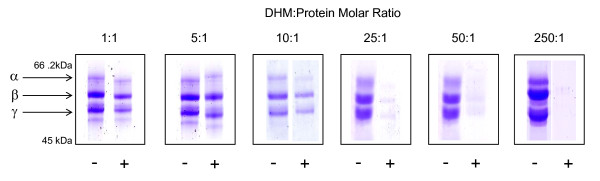
**The effect of pyrrole concentration on fibrinogen standard**. Varying concentrations of DHM were incubated with a bovine fibrinogen standard, which was then analysed using reducing 1-D SDS-PAGE for 120 mins at room temperature. The proteins were separated on 7.5% (w/v) gels and visualised with Coomassie brilliant blue. Higher concentrations of DHM caused the modification of fibrinogen, whereas lower concentrations showed no effect. The α- β- and γ-chains of bovine fibrinogen are labelled. Key: - = control reaction mixture complete apart from DHM; + = reaction mixture containing DHM.

**Figure 4:**
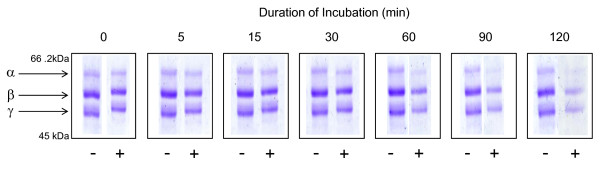
**Time-course of pyrrole modification of fibrinogen standard**. DHM was added to a bovine fibrinogen standard at a 10 molar excess. Aliquots were taken between 0 and 120 min post pyrrole addition and analysed by 1-D SDS-PAGE. Protein samples were mixed with reducing sample buffer and analysed on a 7.5% (w/v) gel. The gel was stained with Coomassie brilliant blue. From 30 min onwards the density of the fibrinogen bands, in particular the α-chain, appear to be diminished after reaction with the pyrrole. Key: - = control reaction mixture complete apart from DHM; + = reaction mixture containing DHM.

Since fibrinogen was reactive with DHM dose-response and time-course experiments were conducted. The optimal concentration for pyrrole exposure was calculated at a molar ratio of DHM:protein of 10:1 (Figure 3). Lower concentrations of the pyrrole failed to completely modify the protein, whereas high concentrations resulted in instantaneous and complete modification. Figure 4 shows 1-D SDS-PAGE analysis of pyrrole-treated fibrinogen over a 120 min incubation period. The density of the fibrinogen bands, and in particular the α-chain, diminished over time, suggesting that the protein is gradually modified in a way which prevents it from entering the gel and migrating to its usual position. This correlates well with the appearance of high molecular weight aggregates.

All of the cysteine residues in fibrinogen maintain the tertiary structure of the protein through disulphide bonds. In principle these oxidised cysteines residues would not be available to react with an alkylating agent such as pyrrole, and modification of amino acid residues other than cysteine may have occurred. Tentative identifications of pyrrole modified peptides were made from MALDI-ToF-MS analysis of in-gel and in-solution tryptic digests of the fibrinogen aggregate, however, additional mass spectrometric analysis using LC-MS/MS was unable to confirm the site pyrrole modification. This may occur due to the fact that the covalent linkage between the pyrrole and amino acid residues may not be stable to conditions of analysis by mass spectrometry [26,27]. The formation of specific pyrrole derived fragment ions can vary depending on the amino acid adducted and the surrounding peptide sequence. Pyrrole modification also disrupts the normal peptide fragmentation [28] complicating the ability to obtain sequence information from modified peptides.

### Western blotting of pyrrole modified plasma proteins

**Figure 5:**
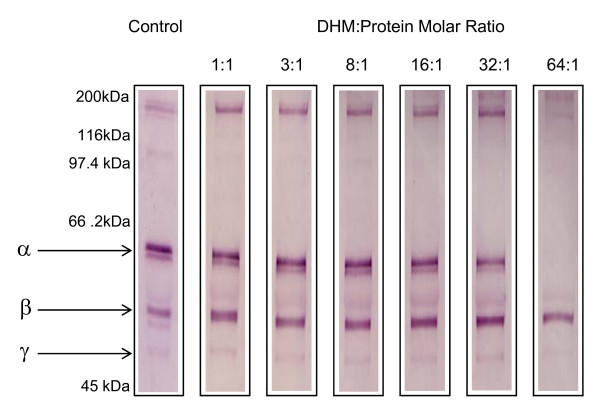
**Monitoring the effect of pyrrole exposure on equine plasma fibrinogen using western blotting**. Equine plasma was reacted with varying concentrations of DHM for 60 min at room temperature. Control plasma was treated with a reaction mixture that was complete apart from DHM. Samples were analysed by 1-D SDS-PAGE and proteins were blotted onto nitrocellulose membranes and blocked overnight before probing with an anti-equine fibrinogen antibody. Blots were then incubated with an alkaline phosphatase linked secondary antibody and developed. Migration of fibrinogen α-chain was evident following the exposure of a large excess of pyrrole.

In order to further investigate the response of plasma proteins with DHM, western blots were performed using antibodies to equine fibrinogen. After exposure to DHM, the fibrinogen α-chain band was no longer observed. Western blots using antibodies to equine serum albumin and transferrin indicated that albumin was modified to a lesser extent following DHM exposure, whereas the transferrin demonstrated no reactivity with pyrrole. A dose-response study revealed that the fibrinogen aggregation is initiated when an approximate sixty-fold molar excess of pyrrole is added to plasma (Figure 5).

## Conclusion

Although ragwort poisoning is recognised as a threat to equidae, the true extent of this threat cannot be assessed in the absence of a definitive and specific test for exposure to the toxin. At present, clinical tests are focused on a more generalised manifestation of hepatic damage. The poisoning is chronic and accumulative, and the emergence of symptoms may post date the exposure to the plant by weeks or even months. There is therefore a pressing need for a test that can assess the history of exposure. The most successful test used previously was based on qualitative assessment of adducts of the pyrrole with blood proteins [14,15]. Accordingly, an *in vitro *model system, not requiring deliberate exposure of horses to pyrrolizidine alkaloids, was developed to explore the specificity of adduction. Perhaps surprisingly, the highly abundant haemoglobin chains were not modified, which might reflect lack of availability of reactive side chains, or the inability of the pyrrole to survive for long enough in plasma to cross the erythrocyte membrane. Plasma proteins might therefore be targets for pyrrole modification, and the discovery that fibrinogen was modified is the first step towards such a specific test.

It is not known whether a protein-based analysis is more sensitive than a test based on measurement of pyrrole residues after release from proteins, or indeed, is sensitive enough for a routine analysis. However, the mass-shifted modification of fibrinogen chains is easily detected by western blotting, and could be readily performed in many laboratories, requiring minimal investment in equipment and using a commercially available antiserum. Because the test is based on a mass shift, a simple ELISA cannot be substituted, as this would not discriminate between the unmodified and modified fibrinogen chains. Deployment of this simple assessment may be of greatest value in identifying and establishing a pool of cases that can then be used to develop even more sensitive test, based on specific peptide modification or on direct detection of pyrrole moieties in a blood sample. Such a screening test may enable veterinarians to identify ragwort exposure and would therefore be an invaluable diagnostic tool with substantial welfare benefits.

## Methods

### Experimental samples

Whole blood was collected into lithium heparin tubes from horses by venepuncture as part of routine clinical investigations. Any surplus blood (approximately 10 mL) was used in this study. The blood was spun at 1,300 × *g *for 10 min and the plasma removed. Erythrocytes were then washed three times with isotonic phosphate buffered saline (10 mM phosphate, 140 mM NaCl, 2.6 mM KCl, pH 7.4) and lysed by adding five volumes of distilled water. Erythrocyte membranes were removed by centrifuging the haemolysate at 18,000 × *g *for 20 min.

Ethical statement: The study was performed in adherence to the University of Liverpool Animal Ethics Guidelines.

### Chemical synthesis of DHM

DHM was synthesised by the chemical oxidation of monocrotaline [29,30]. Monocrotaline (10 mg, Sigma, Poole, UK) dissolved in 2 mL chloroform was added to 21 mg tetrabromo-1,2-benzoquinone (*o*-bromanil) (Sigma) dissolved in 2 mL chloroform and stirred for 2 min at room temperature. A mixture of sodium borohydride (3 mg) and sodium hydroxide (700 mg) dissolved in 2 mL distilled water was added to quench the reaction, resulting in a green precipitate. The lower organic layer was immediately collected, dried over anhydrous sodium sulphate, filtered through decolorising charcoal and concentrated to dryness under nitrogen gas. The DHM was then redissolved in DMF and used immediately. The approximate yield of DHM was 95–100%.

### Reaction of pyrrole with proteins

The protein concentration of haemoglobin and plasma was determined using the Coomassie Plus Protein Assay (Pierce Biotechnology, Rockford, USA). To haemoglobin lysate (approximately 25 mg) or plasma (approximately 10 mg), DHM dissolved in DMF was added in molar ratios of between 1:1 and 100:1 DHM:protein. This was left mixing at room temperature for 60 min. Bovine fibrinogen, serum albumin and transferrin protein standards (Sigma) were dissolved at 1 mg/mL in 50 mM ammonium bicarbonate. DHM was added to a final molar ratio of 10:1 DHM:protein and left to react at room temperature for up to 120 min. The reaction of pyrrole with protein was halted at each time point by addition of an equal volume of 1 mg/mL glutathione in 50 mM ammonium bicarbonate. Multiple control plasma samples were analysed as follows: plasma treated with the reaction mixture without DHM; plasma treated with monocrotaline alone; and plasma treated with the carrier solvent, DMF. The approximate molecular weight used for calculating the molar ratios of DHM incubations of complete plasma was 55 kDa. Replicate experiments were performed on five separate occasions.

### 1-D SDS-PAGE

Plasma samples and protein standards (10 μg) were resolved at a constant potential of 200V through either 7.5 or 12.5% (w/v) polyacrylamide gels with 4% (w/v) stacking gel. The samples were boiled at 100°C for 5 min in a reducing buffer (125 mM Tris-HCl; 140 mM SDS; 20% (v/v) glycerol; 200 mM dithiothreitol (DTT) and 30 mM bromophenol blue) or left at room temperature for 5 min in a non-reducing buffer (as for reducing buffer minus DTT) prior to loading. Gels were stained with Coomassie brilliant blue (Amersham Biosciences, Buckinghamshire, UK).

### Digestion of proteins with trypsin

Gel plugs from protein bands of interest were excised and the proteins were destained by repeated incubation in a solution of 50 mM ammonium bicarbonate in 50% (v/v) acetonitrile at 37°C for 20 min. When preparing samples for the detection of pyrrole modifications the gel plugs were dehydrated with acetonitrile and allowed to air dry. For protein identification, the plugs were additionally washed in 50 mM ammonium bicarbonate and incubated in 10 mM DTT at 37°C. After 30 min the supernatant was discarded, 55 mM iodoacetamide was added and the incubation was continued in the dark for 60 min. In all cases, gel plugs were digested with 10 μL of trypsin (10 ng/μL) in 50 mM ammonium bicarbonate. In-solution digestion of protein samples was performed in 50 mM ammonium bicarbonate with trypsin (10 μg/μL) at a ratio of protein:trypsin 50:1.

### Intact mass analysis by ESI-MS

Intact mass analysis of haemoglobin was performed using ESI-MS on a Q-ToF Micro tandem mass spectrometer (Waters, Manchester, UK), equipped with a nanospray source. All analyses were performed in positive ion mode. Proteins were diluted to a concentration of 1 μM with acetonitrile/water (50/50, v/v) containing 0.1% (v/v) formic acid and directly infused into the ESI source at a flow rate of 0.5 μL/min. Data were acquired between *m/z *500–2000. Typically 20 scans were combined into a single spectrum, which was subsequently deconvoluted using MaxENT 1 maximum entropy software (Waters). Mass spectra were processed between 15,000 and 17,000 Da at 1 Da/channel.

### Peptide mass fingerprinting by MALDI-ToF-MS

Peptide analysis of protein (haemoglobin) digestion products was performed in positive ion mode on a M@LDI R mass spectrometer (Waters, Manchester, UK). The instrument was calibrated for each batch of analysis using a standard mix containing *des*-arg bradykinin (*M*_r _903.47), neurotensin (*M*_r _1671.92), adrenocorticotrophic hormone (*M*_r _2464.20) and insulin-β chain (*M*_r _3493.65). All standards were purchased from Sigma (Poole, UK). Samples were mixed 1:1 with a saturated solution of α-cyano-4-hydroxy cinnamic acid in 50% v/v ACN 0.1% v/v trifluroacetic acid and 2 μL of the resultant mixture were spotted onto a MALDI target. MALDI spectra were acquired over the *m/z *range 800–4000. Proteins were identified from their peptide mass fingerprints by manual searching using a locally implemented MASCOT server version 1.9  against the MSDB and SWISS-PROT databases. Initial search parameters allowed a mass tolerance of ± 250 ppm, a variable trypsin missed cleavage, carbamidomethyl modification of cysteine residues and variable oxidation of methionine. The taxonomic search space was restricted to *Chordata*.

### LC-MS/MS analysis

Plasma proteins were identified by LC-MS/MS analysis of peptide digests using an LTQ ion-trap mass spectrometer (Thermo-Electron, Hemel Hempstead, UK) equipped with a nanospray electrospray ionisation source coupled to a Ultimate 3000 nanoLC system (Dionex, Camberley, UK). The LTQ was calibrated using a 500 fmol/μL solution of human [Glu^1^]-fibropeptide B (*M*_r _1570.62). The samples were initially desalted and concentrated on a C18 PepMap pre-column (5 mm × 300 μm i.d.) (Dionex) prior to separation on a C18 PepMap column (15 cm × 75 μm i.d.) (Dionex). The column was equilibrated in 0.1% (v/v) formic acid (FA) at a flow rate of 0.3 μl/min. Peptides (20 μL) were then loaded and washed for 5 min at the same flow rate prior to being eluted with a linear gradient of 0–100% (v/v) acetonitrile/0.1% (v/v) FA over 50 min at a flow rate of 0.2 μL/min. All analyses were in positive ion mode. MS/MS spectra were acquired in the data dependant mode. Doubly or triply charged peptide ions were identified from a survey scan (*m/z *400–1500), following which the top three individual precursor ions were automatically selected for fragmentation using the "triple-play" mode. The spectral data were converted to DTA files using the SEQUEST search engine. The merged DTA files were then used to search a locally implemented MASCOT server version 1.9 against the MSDB databases. Search parameters allowed a peptide tolerance of ± 200 ppm, MS/MS tolerance of ± 0.8 Da, up to one missed trypsin cleavage and with carbamidomethylation of cysteine residues and oxidation of methionine as variable modifications. The taxonomic search space was restricted to *Chordata*.

### Western blotting

The identity of proteins was confirmed using western blotting. Equine plasma was incubated with DHM as described above and the proteins were separated using 1-D SDS-PAGE. The proteins were transferred to a 0.2 μm nitrocellulose membrane (Schleicher & Schuell, Dassel, Germany) at 200 mA for 120 min. The blot was probed with a 1:50,000 dilution of rabbit (polyclonal) anti-(equine) fibrinogen (Affiland, Belgium) for 120 min at room temperature. To detect transferrin and albumin in equine plasma rabbit anti-equine transferrin and rabbit anti-equine albumin antibodies (Bethyl Laboratories, Cambridge, UK) were used respectively. Each blot was incubated for 60 min with a 1:5000 dilution of goat anti-rabbit IgG (whole molecule) alkaline phosphatase-conjugated secondary antibody (Sigma). The blot was developed with 5-bromo-4-chloro-3-indolyl phosphate/nitro blue tetrazolium until colour developed, after which the substrate was decanted and the blots rinsed with water.

## Authors' contributions

REM performed the pyrrole modification experiments, protein characterisation analysis and co-wrote the manuscript. DCK participated in the design of the study, obtained the horse blood samples and contributed to the manuscript drafting. JBM contributed to the study design and revision of the manuscript. RJB conceived the study, participated in the data interpretation and critically evaluated the manuscript. PDW managed the study, contributed to the interpretation of the data and led the manuscript preparation. All authors read and approved the final manuscript.
